# Persistent viral shedding of SARS‐CoV‐2 in faeces – a rapid review

**DOI:** 10.1111/codi.15138

**Published:** 2020-06-04

**Authors:** S. Gupta, J. Parker, S. Smits, J. Underwood, S. Dolwani

**Affiliations:** ^1^ Division of Population Medicine Cardiff University School of Medicine Cardiff UK; ^2^ Division of Infection and Immunity Department of Infectious Diseases Cardiff and Vale University Health Board Cardiff University Cardiff UK

**Keywords:** COVID‐19, faeces, gastrointestinal, SARS‐CoV‐2, viral shedding

## Abstract

**Aim:**

In addition to respiratory symptoms, COVID‐19 can present with gastrointestinal complaints suggesting possible faeco‐oral transmission. The primary aim of this review was to establish the incidence and timing of positive faecal samples for SARS‐CoV‐2 in patients with COVID‐19.

**Methods:**

A systematic literature review identified studies describing COVID‐19 patients tested for faecal virus. Search terms for MEDLINE included ‘clinical’, ‘faeces’, ‘gastrointestinal secretions’, ‘stool’, ‘COVID‐19’, ‘SARS‐CoV‐2’ and ‘2019‐nCoV’. Additional searches were done in the *American Journal of Gastroenterology*, *Gastroenterology*, *Gut*, *Lancet* *Gastroenterology and Hepatology*, the World Health Organization Database, the Centre for Evidence‐Based Medicine, *New England Journal of Medicine*, social media and the National Institute for Health and Care Excellence, bioRxiv and medRxiv preprints. Data were extracted concerning the type of test, number and timing of positive samples, incidence of positive faecal tests after negative nasopharyngeal swabs and evidence of viable faecal virus or faeco‐oral transmission of the virus.

**Results:**

Twenty‐six relevant articles were identified. Combining study results demonstrated that 53.9% of those tested for faecal RNA were positive. The duration of faecal viral shedding ranged from 1 to 33 days after a negative nasopharyngeal swab with one result remaining positive 47 days after onset of symptoms. There is insufficient evidence to suggest that COVID‐19 is transmitted via faecally shed virus.

**Conclusion:**

There is a high rate of positive polymerase chain reaction tests with persistence of SARS‐CoV‐2 in faecal samples of patients with COVID‐19. Further research is needed to confirm if this virus is viable and the degree of transmission through the faeco‐oral route. This may have important implications on isolation, recommended precautions and protective equipment for interventional procedures involving the gastrointestinal tract.

## Introduction

The rapid progression of the COVID‐19 pandemic has created significant challenges for the public as well as healthcare professionals around the world. Knowledge regarding virus incubation, transmission and shedding is crucial for the reduction of new cases and protection of healthcare professionals. Guidance regarding isolation and protective equipment has changed as evidence has increased and developed.

The high incidence of cough and fever in COVID‐19 is well established [1]. Gastrointestinal symptoms are also well documented suggesting a potential faeco‐oral transmission route [2]. Discharge guidelines for hospitals for declaring a COVID‐19 patient recovered in the UK are largely based on time from either symptom onset or positive test depending on the severity of illness and the discharge destination [3].The European Centre for Disease Prevention and Control, on the other hand, has advocated the need for continued self‐isolation and hand hygiene measures even 14 days post‐discharge based on prolonged viral shedding in faeces and respiratory samples [4]. This evidence may influence the recommended duration of self‐isolation, home sanitation practices during isolation and after discharge and the use of protective equipment for procedures involving the gastrointestinal tract. Evidence‐based recommendations for specialities such as gastroenterology, gastrointestinal endoscopy and gastrointestinal surgery are required where there may be an exposure risk to virus shed in faeces. Despite viral RNA being detected in the air or other surface samples like toilets, it is still unclear whether it is viable to transmit infection through this route [5].

The primary aim of this review is to assess the incidence and timing of positive faecal samples for SARS‐CoV‐2 in relation to the clinical course of patients with COVID‐19.

Our secondary aims are to establish the incidence of patients with positive faecal samples after negative respiratory swabs and any evidence to suggest faecal virus transmitted infection.

## Method

Reports of cases or studies of COVID‐19 patients with evidence of the virus in faecal samples were systematically identified and full text articles were reviewed for data extraction.

### Literature search

A comprehensive search was undertaken as per the search strategy outlined below for literature that included SARS‐CoV‐2 virus testing of faeces. MEDLINE was searched to find articles published until 3 April 2020. The defined search terms were created after collaboration between the authors experienced in gastroenterology, colorectal surgery and systematic review. Search terms reflected the aim to identify studies with evidence of faecal COVID‐19 and included ‘clinical’, ‘faeces’, ‘gastrointestinal secretions’, ‘stool’, ‘COVID‐19’, ‘SARS‐CoV‐2’ and ‘2019‐nCoV’. Additional manual searches to identify the most recent evidence were performed in the *American Journal of Gastroenterology*, *Gastroenterology*, *Gut*, the *Lancet Gastroenterology and Hepatology*, the World Health Organization (WHO) Database, the Centre for Evidence‐Based Medicine, the *New England Journal of Medicine* and the National Institute for Health and Care Excellence. COVID‐19 preprints published until 10 April 2020 on medRxiv and bioRxiv and an independent search on social media (Twitter) by the authors (SS, SD) added more articles. The search strategy used for social media and a brief description of the WHO and other databases are provided in Appendix S1.

### Inclusion and exclusion criteria

Articles describing COVID‐19 patients who had faecal or stool specimens tested for the virus were included. Considering the knowledge gaps existing for COVID‐19 all articles were considered regardless of the number, age or gender of patients or the country of publication. Animal‐based studies or articles without an available full text were excluded. Foreign language articles were considered but excluded unless the necessary language expertise was available within the research group.

### Study identification

Articles were sorted alphabetically by author name and divided between two reviewers (SG and JP). Abstracts were reviewed and classified by the same two authors through the Rayyan Web Application [6] to identify those for full text review. The same process was used for full text articles and these data were managed through EndNote (EndNote X9.3.1 license provided by Cardiff University). Articles were then discussed between the same reviewers to identify the final selection of full text articles. Any conflicts were solved by the supervising author if necessary. Reference lists and review articles were cross‐referenced to identify any further original studies. All articles were categorized and described in a PRISMA flow chart.

### Data extraction

The final data extraction was also carried by the two reviewers (JP and SG) and managed through Microsoft Excel files. The data parameters extracted from the studies are shown in Table 1. The final data were verified by the two reviewers (JP and SG) with conflict resolution as described previously if necessary.

**Table 1:** Data parameters for extraction.

1.	Study reference
2.	Country of publication
3.	Number and type of patients in the study
4.	Type of sample taken (faecal sample, anal swab, RT‐PCR, culture)
5.	Number of patients having faecal samples tested and number of positive samples
6.	Timing of positive faecal swab after symptom onset
7.	Duration of positive faecal specimen after negative nasopharyngeal swab
8.	Any evidence for viable faecal virus or faeco‐oral transmission documented in the study

## Results

**Figure 1:**
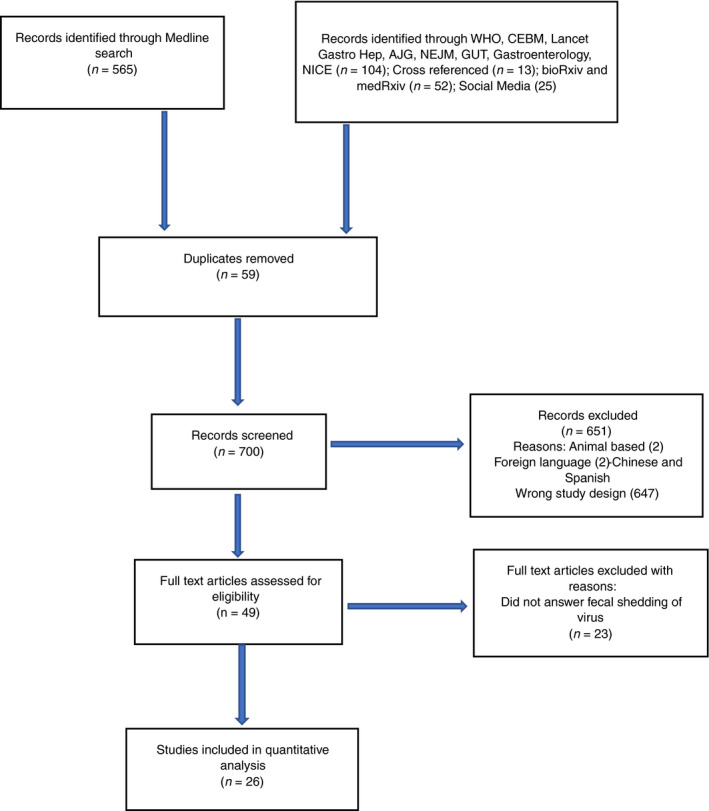
PRISMA flow chart.

MEDLINE searches identified 565 articles and 194 were found through other databases. An overview of the selection process is shown in the PRISMA chart in Fig. 1. There were 26 articles [7, 8, 9, 10, 11, 12, 13, 14, 15, 16, 17, 18, 19, 20, 21, 22, 23, 24, 25, 26, 27, 28, 29, 30, 31, 32] included in the final analysis. An overview of the patient demographics is summarized in Table 2.

**Table 2:** Overview of patient demographics from studies included in the review [7, 8, 9, 10, 11, 12, 13, 14, 15, 16, 17, 18, 19, 20, 21, 22, 23, 24, 25, 26, 27, 28, 29, 30, 31, 32].

Reference	Country	Number of patients in study	Type of patients	Type of sample
Cai *et al*. [7]	China	10	Children, 3–131 months	Faeces
Chan *et al*. [8]	China	6	Family cluster (10–66 years)	Faeces
Chen *et al*. [9]	China	1	Man, 34 years	Faeces
Chen *et al*. [10]	China	1	Woman, 25 years	Faeces
Chen *et al*. [11]	China	57	Unclear	Anal swab
Han *et al*. [12]	China	206	Adults	Faeces
Holshue *et al*. [13]	USA	1	Man, 35 years	Faeces
Kim *et al*. [14]	Korea	2	Adult: man and woman	Faeces
Kujawski *et al*. [15]	USA	12	Adults	Faeces
Lescure *et al*. [16]	France	5	Adults	Faeces
Ling *et al*. [17]	China	66	Adults	Faeces
Lo *et al*. [18]	China	10	9 adults, 1 child	Faeces
Nicastri *et al*. [19]	Italy	1	Adult, late 20s	Faeces
Pan *et al*. [20]	China	17	Laboratory samples	Faeces
Peng *et al*. [21]	China	9	Adults	Anal swab
Song *et al*. [22]	China	1	Middle aged woman	Anal swab
Tan *et al*. [23]	China	1	Man, 73 years	Rectal swab
Tang *et al*. [24]	China	1	Man, 10 years	Faeces
Wang *et al*. [25]	China	205	Adults and children, mean age 44 years	Faeces
Wu *et al*. [26]	China	74	Laboratory samples	Faeces
Xiao *et al*. [27]	China	73	Children and adults, 10 months to 78 years old	Faeces
Xing *et al*. [28]	China	3	Children, 1.5–6 years	Faeces
Xu *et al*. [29]	China	10	Children, 2 months to 15 years	Rectal swab
Zhang *et al*. [30]	China	23	Adults, median age 48 years	Faeces
Zhang *et al*. [31]	China	14	Adults, median age 41 years	Faeces
Zhang *et al*. [32]	China	15	Laboratory samples	Anal swab

Most studies were from China (*n* = 20) with two from the USA and one each from Italy, Korea, Vietnam and France. The number of participants recruited in the studies ranged from 1 to 206 with ages ranging from 3 months to 87 years. Sample collection consisted of faecal samples or anal or rectal swabs. Quantitative reverse transcription polymerase chain reaction (RT‐PCR) was the test performed on all samples to detect viral RNA.

The indication for faecal testing was not specified in most studies. In some the test was done in asymptomatic patients for screening after contact with an infected person or travel history to an infected area. The predominant symptoms of presentation in the studies were persistent cough, fever and breathlessness with fewer patients reporting diarrhoea or vomiting. All studies had information regarding our primary aim of reporting faecal samples for the virus in those with COVID‐19. Of these, 16 [7, 10, 11, 14, 15, 16, 17, 18, 19, 23, 24, 26, 27, 28, 29, 30] provided information on the duration of these tests after symptom onset and evidence of positive faecal samples after symptom recovery, discharge from the hospital or negative nasopharyngeal RT‐PCR. The data extraction is summarized in Tables 3 and 4 which are divided based on the number of patients tested for faecal RT‐PCR in the study (≤ 10 and > 10 respectively) and the detailed combined table is attached as supplementary results (Table S1).

**Table 3:** Overview of data extracted from studies included in the review with ≤ 10 patients tested for faecal virus [7, 8, 9, 10, 13, 14, 15, 16, 18, 19, 21, 22, 23, 24, 28, 29]

Reference	Patients with positive faecal RT‐PCR	Timing of positive faecal RT‐PCR (from symptom onset unless stated otherwise)	Number of patients with positive faecal RT‐PCR and negative NP RT‐PCR	Duration of persistent positive faecal RT‐PCR after negative NP RT‐PCR
Cai *et al*. [7]	6 tested, 5 positives (83.3%)	First test at 3–13 days Second test at 18–30 days Positive in all patients on both tests	5 out of 5 (100%)	Ranged from 11 to 18 days
Chan *et al*. [8]	4 tested, 0 positive	NA	NA	NA
Chen *et al*. [9]	1 tested, 0 positive	NA	NA	NA
Chen *et al*. [10]	1 tested, 1 positive (100%)	Day 11	1 out of 1 (100%)	1 day
Holshue *et al*. [13]	1 tested, 1 positive (100%)	Day 7	Not available	Not available
Kim et al. [14]	2 tested, 2 positives (100%)	Ranged from day 8 to 17	0 out of 2	NA
Kujawski *et al*. [15]	10 tested, 7 positives (70%)	Ranged from day 6 to 18	2 out of 7 (28.6%)	Ranged from 4 to 6 days
Lescure *et al*. [16]	5 tested, 2 positives (40%)	Ranged from day 2 to 13	1 out of 2 (50%)	3 days
Lo *et al*. [18]	10 tested, 10 positives (100%)	Ranged from day 2 to 19	4 out of 10 (40%)	Ranged from 2 to 10 days
Nicastri *et al*. [19]	1 tested, 1 positive (100%)	Day 3 after admission	0 out of 1	NA
Peng *et al*. [21]	9 tested, 2 positives (22.2%)	Patient 1: day 3 Patient 2: unknown	Not available	Not available
Song *et al*. [22]	1 tested, 0 positive	NA	NA	NA
Tan *et al*. [23]	1 tested, 1 positive (100%)	Up to day 23	1 out of 1 (100%)	7 days
Tang *et al*. [24]	1 tested, 1 positive (100%)	Ranged from day 17 to 25 after exposure	1 out of 1 (100%)	10 days
Xing *et al*. [28]	3 tested, 3 positives (100%)	Patient 1 and 2: day 4 Patient 3: day 9 (after discharge)	3 out of 3 (100%)	8 and 20 days
Xu *et al*. [29]	10 tested, 8 positives (80%)	Ranged from day 1 to 3	8 out of 8 (100%)	Ranged from 3 to 21 days

NA, not applicable; NP, nasopharyngeal; RT‐PCR, reverse transcription polymerase chain reaction.

**Table 4:** Overview of data extracted from studies included in the review with > 10 patients tested for faecal virus [11, 12, 17, 20, 25, 26, 27, 30, 31, 32].

Reference	Patients with positive faecal RT‐PCR	Timing of positive faecal RT‐PCR (from symptom onset unless stated otherwise)	Number of patients with positive faecal RT‐PCR and negative NP RT‐PCR	Duration of persistent positive faecal RT‐PCR after negative NP RT‐PCR
Chen *et al*. [11]	28 tested, 11 positives (39.3%)	Only specify timings in two patients Patient 1: day 13 Patient 2: day 10	1 out of 2 (50%)	3 days
Han *et al*. [12]	22 tested, 12 positives (54.5%)	Not available	Not available	Not available
Ling *et al*. [17]	66 tested, 66 positives (100%)	Not available	43 out of 66 (65%)	Duration to negative NP sample ranged from 6 to 11 days (median 9.5 days) *vs* Duration to negative faecal sample ranged from 9 to 16 days (median 11 days) NB: 11 patients still had positive faecal RT‐PCR at 31 days after admission to convalescence
Pan *et al*. [20]	17 tested, 9 positives (53%)	Ranged from day 0 to 11	Not available	Not available
Wang *et al*. [25]	153 tested, 44 positives (29%)	Not available	Not available	Not available
Wu *et al*. [26]	74 tested, 41 positives (55%)	Variable	32 out of 41 (78%)	Faecal sample remained positive for a mean duration of 27.9 days (9.2 days longer than positive respiratory sample) Patient 1: 33 days after negative nasopharyngeal swab Patient 2: 47 days from symptom onset
Xiao *et al*. [27]	73 tested, 39 positives (53.4%)	Ranged from day 1 to 12 days	17 out of 39 (23.3%)	Not available
Zhang *et al*. [30]	12 tested, 10 positives (83.3%)	Day 4	6 out of 10 (60%)	Median duration of positive NP sample 10 days *vs* median duration of positive faecal sample 22 days
Zhang *et al*. [31]	14 tested, 5 positives (35.7%)	Ranged from day 4 to 10	Not available	Not available
Zhang *et al*. [32]	15 tested, 4 positives (26.7%)	Ranged from day 0 to 5	Not available	Not available

NA, not applicable; NP, nasopharyngeal; RT‐PCR, reverse transcription polymerase chain reaction.

A total of 824 patients were included across the studies and 540 were tested for faecal viral RNA [7, 8, 9, 10, 11, 12, 13, 14, 15, 16, 17, 18, 19, 20, 21, 22, 23, 24, 25, 26, 27, 28, 29, 30, 31, 32]. Positive faecal RT‐PCR tests occurred in 291 (53.9%). The timing of the first positive sample was available in 21 studies and varied from day 0 of symptom onset to day 17. Late positive tests do not necessarily equate to absence of the virus earlier in the illness but may reflect the heterogeneity in testing patterns amongst the studies. First stool samples were often reported late after hospital admission [11] or even after discharge [28] while some were analysed from day 1 of hospitalization or symptom onset [19, 20, 27, 29, 32]. There is a similar discrepancy in follow‐up testing. Some tested until samples were found to be negative [17] while others did not [18, 29].

Of 199 patients who tested positive for faecal viral RNA and who were followed up with stool testing, 125 (62.8%) showed persistent shedding of virus in the stool samples after a negative nasopharyngeal swab while in the individual studies it ranged from 23.3% to 100%. The duration for faecal shedding of viral RNA after clearance of respiratory samples ranged from 1 to 33 days and in one patient up to 47 days from symptom onset [26].

None of the studies was designed to detect live virus in the faeces except for the study by Wang *et al*. [25]. Of 153 stool specimens tested in this study, 44 were PCR positive and, of four specimens cultured, live virus was detected in two [25].

## Discussion

This rapid review demonstrates a high incidence and persistence of positive faecal RT‐PCR tests for SARS‐CoV‐2 after negative nasopharyngeal swabs in patients with COVID‐19. This may have important implications regarding measures to prevent the spread of disease, precautions recommended for the public and protective equipment for health professionals performing interventions involving the gastrointestinal tract.

A Chinese review performed by Tian *et al*. [33] summarized evidence on the importance of identifying gastrointestinal symptoms in addition to the respiratory symptoms of patients with COVID‐19. Despite persistent shedding of SARS‐CoV‐2 virus in faeces there seems to be no correlation with the presence or severity of gastrointestinal symptoms based on the limited data available. Our review adds to this evidence from China and describes the plausibility of faeco‐oral transmission.

Despite this review demonstrating a high incidence of positive tests for virus in the faeces, the absence of evidence to confirm infectivity from this must be emphasized. In order to adequately confirm this, good quality evidence is required to demonstrate infectious virus in faeces and its risk of transmitting disease between individuals. These data may then enable the development of reliable guidelines and recommendations. However, given the rapid development of the pandemic, this will take time and reviews such as this may help guide focused and valuable research questions for the future. The findings of our review provide a synopsis of the best available evidence regarding SARS‐CoV‐2 in the faeces at the current time.

Evidence regarding other coronaviruses may be helpful in this context. Similar patterns of virus isolation from stool and faeco‐oral transmission were observed for other coronaviruses including SARS‐CoV‐1 [34]. Bio‐aerosol generation of viral particles as a result of toilet flushing, the impact of disinfection on this [35, 36] and the persistence of coronaviruses on surfaces has been studied before [37]. Other indirect evidence of microbial exposure and contamination of the operator’s face during endoscopy [38] and laboratory evidence of SARS‐CoV‐2 infection of the gastrointestinal tract and mechanisms [39, 40] add to the evidence for plausibility of transmission.

The risk to healthcare professionals from patient exposure is well known, specifically in high aerosol generating procedures. Professional societies and investigator groups from countries with experience of managing COVID‐19 in the context of gastrointestinal interventions [41, 42] highlight the risk to individuals in endoscopy departments and the need for necessary precautions including negative pressure rooms and personal protective equipment for both upper and lower gastrointestinal procedures. This review supports the importance of these measures given a high prevalence and persistence of SARS‐CoV‐2 virus in faeces. Isolation of live virus is confirmed only by one study [25] and the proportion of cases that might be transmitted by this route is unclear due to the heterogeneity in case selection and lack of standardization of study designs and protocols. Environments such as care homes may be particularly vulnerable to transmission of infection by this route and recommendations must take into account this evidence to ensure the protection of health and social care providers and the general public in the meantime. Application of these data to the population may be helpful in guiding the recommendations for isolation periods to reduce transmission rates.

### Limitations

Despite finding a high incidence of positive faecal samples for SARS‐CoV‐2 in the included studies, our review cannot confirm the true population prevalence of positive faecal samples or the rate of false negatives. This is due to the significant variability in study design which is an inherent problem with COVID‐19 research at present. This heterogeneity was not formally assessed due to it being a rapid review but can be clearly identified on inspection of the study designs and outcomes. The variability in patient numbers and characteristics, sample timing, sample nature (faecal samples *vs* anal or faecal swabs) and follow‐up testing should be considered when interpreting the reliability of the results. If other studies confirm viable virus in stool, then methods of culture also need to be described and standardized for comparison and replication in other populations. The majority of the included studies are small, heterogeneous, retrospective and often did not assess viral shedding in the faeces as their primary aim. At present, however, this is the only evidence available. There were two foreign language articles excluded due to lack of translation resources. The preprints are not peer reviewed and therefore should be treated with caution.

## Conclusion

The duration of viral shedding in the faeces is mostly reported from 1 to 33 days after a negative nasopharyngeal swab but can continue for up to 47 days after onset of symptoms in patients with COVID‐19. These positive samples can occur after negative nasopharyngeal swabs or resolution of patient symptoms. Isolation of live virus in stool specimens of two cases in a single study supports the possibility of faeco‐oral transmission. Further research is needed to prove whether this viral shedding in stool results in a significant proportion of case transmissions in the community as well as within care institutions and secondary care. Until further evidence is generated appropriate precautions should be recommended for the protection of healthcare workers and patients.

## Implications for the public

1. In addition to strict adherence to hand washing recommendations, home toilet sanitary and disinfection precautions should be taken in the case of isolation or contact with a symptomatic COVID‐19 case with or without gastrointestinal symptoms. This statement is based on limited evidence of possible viable faecal virus excretion.
2. These precautions may need to continue for longer than the period of symptoms and the current recommendations for isolation after symptoms cease. This statement is based on limited evidence of the duration after the onset of symptoms that an RT‐PCR stool test might still be positive.

## Implications for healthcare professionals

1. Professional bodies’ recommendations on protective equipment, endoscopic and surgical procedures for COVID‐19 patients should be followed [43, 44, 45, 46].
2. The possibility of faeco‐oral transmission should be borne in mind with implications for endoscopy and theatre disinfection of surfaces in between procedures.
3. Ward areas for COVID‐19 patients and care homes or similar institutions may need to consider the implications for infection control and disinfection in the light of the possibility of faeco‐oral transmission.
4. Screening processes for patients due to undergo investigational or interventional procedures may need to consider including gastrointestinal symptoms and stool testing in future pre‐procedure questionnaires.
5. Healthcare teams managing patients with gastrointestinal symptoms may need to consider the possibility of COVID‐19 coexisting with or worsening symptoms of underlying conditions such as inflammatory bowel disease [47].

## Recommendations for further research

1. Future studies on viral shedding and infectivity of SARS‐CoV‐2 should consider standardization of sampling methods in terms of the timing and the type of sample collection, with appropriate precautions for laboratory staff handling these samples until the situation is clearer.
2. Study designs may wish to consider repeat and parallel sampling with nasopharyngeal swabs at defined time points. This may be correlated with symptoms and serology to clarify the effect of neutralizing antibodies and viable virus excretion in the stool.
3. Study designs may benefit from testing stool samples from comparable groups. This could include symptomatic, asymptomatic or recovered individuals in and out of family clusters and with or without gastrointestinal symptoms. This may improve our understanding of clinical and public health implications and potential targets for intervention in these settings.

## Sources of funding

S Gupta is funded by the Wales Cancer Research Centre and Cardiff University. J Parker is funded by a Royal College of Surgeons of England Research Fellowship. S Smits is funded by Health and Care Research Wales health fellowship (ref. HF‐17‐1352) J Underwood is funded by the Medical Research Council (Grant ref: MR/T023791/1). S Dolwani is Chief Investigator of the CONSCOP2 study funded by the NIHR (HTA Project: NIHR127914).

## Conflicts of interest

None declared.

## Author contributions

SG, JP contributed to conception and design of the project, data collection, analysis and interpretation, drafting of the article, revisions and final approval. SS contributed to data collection, review of the manuscript and final approval of the article. JU contributed to data collection, interpretation, review of the manuscript and final approval of the article. SD contributed to the conception and design of the project, data collection, revisions and final approval of the article and was overall supervisor.

## Supporting information


**Table S1.** Overview of data extracted from studies included in the review [7–32].

**Appendix S1.** Supplementary methods.
