# Supplementary material for: Persistent viral shedding of SARS‐CoV‐2 in faeces – a rapid review
Source: Colorectal Dis. 2020 Jun 4;22(6):611–20. doi: 10.1111/codi.15138 (PMC7276890; doi:10.1111/codi.15138)
Supplement: Supplementary file 1 — Table S1. Overview of data extracted from studies included in the review [7–32]. [file CODI-22-611-s001.docx]

**Supplementary results table**

| **Reference** | **Country** | **Number of patients in study** | **Type of patients** | **Patient symptoms** | **Type of sample** | **Patients with positive faecal RT-PCR** | **Timing of positive faecal RT-PCR**  **(from symptom onset unless stated otherwise)** | **Number of patients with positive faecal RT-PCR and negative NP RT-PCR** | **Duration of persistent positive faecal RT PCR after negative NP RT-PCR** |
| --- | --- | --- | --- | --- | --- | --- | --- | --- | --- |
| **Cai et al. [7]** | China | 10 | Children, 3 to 131 months | Fever  Respiratory symptoms | Faeces | 6 tested, 5 positives (83.3%) | First test at 3-13 days  Second test at 18-30 days  Positive in all patients on both tests | 5 out of 5 (100%) | Patient 1: 18 days; Patient 3: 12 days; Patient 4: 11 days; Patient 5: 12 days; Patient 7: 15 days |
| **Chan et al. [8]** | China | 6 | Family cluster (10 to 66 years) | Fever  Respiratory symptoms Digestive symptoms (2 patients) | Faeces | 4 tested, 0 positive | NA | NA | NA |
| **Chen et al. [9]** | China | 1 | Male, 34 years | Fever  Respiratory symptoms | Faeces | 1 tested, 0 positive | NA | NA | NA |
| **Chen et al. [10]** | China | 1 | Female, 25 years | Fever  Respiratory symptoms | Faeces | 1 tested, 1 positive (100%) | Day 11 | 1 out of 1 (100%) | 1 day |
| **Chen et al. [11]** | China | 57 | Unclear | Respiratory symptoms | Anal swabs | 28 tested, 11 positives (39·3%) | Only specify timings in two patients  Patient 1: day 13  Patient 2: day 10 | 1 out of 2 (50%) | Patient 1: 3 days |
| **Han et al. [12]** | China | 206 | Adults | Matched case series of three groups: 1. Mild respiratory symptoms 2. Mild respiratory and digestive symptoms 3. Only digestive symptoms | Faeces | 22 tested, 12 positives (54·5%) | Not available | Not available | Not available |
| **Holshue et al. [13]** | USA | 1 | Male, 35 years | Fever  Respiratory symptoms Digestive symptoms | Faeces | 1 tested, 1 positive (100%) | Day 7 | Not available | Not available |
| **Kim et al. [14]** | Korea | 2 | Adult male and female | Fever  Respiratory symptoms Digestive symptoms (1 patient) | Faeces | 2 tested, 2 positives (100%) | Patient 1: day 8-12  Patient 2: day 17 | 0 out of 2 | NA |
| **Kujawski et al. [15]** | USA | 12 | Adults | Fever  Respiratory symptoms Digestive symptoms (1 patient) | Faeces | 10 tested, 7 positives (70%) | Patient 1: days 11-18  Patient 2: days 11-18  Patient 3: day 7  Patient 4: days 10-14  Patient 5: days 6-13  Patient 6: days 14-18  Patient 7: days 6-11 | 2 out of 7 (28·6%) | 4 to 6 days |
| **Lescure et al. [16]** | France | 5 | Adults | Fever  Respiratory symptoms Digestive symptoms (1 patient) | Faeces | 5 tested, 2 positives (40%) | Patient 1: day 2-9  Patient 2: day 3-13 | 1 out of 2 (50%) | Patient 1: 0 day  Patient 2: 3 days |
| **Ling et al. [17]** | China | 66 | Adults | Unknown | Faeces | 66 tested, 66 positives (100%) | Not available | 43 out of 66 (65%) | Median duration to negative NP RT-PCR:  9.5 days (range 6-11)  Median duration to negative faecal RT-PCR:  11 days (range 9-16)  N.B.: 11 patients still had positive faecal RT-PCR at 31 days after admission to convalescence |
| **Lo et al. [18]** | China | 10 | 9 adults, 1 child | Fever Respiratory symptoms Digestive symptoms (8 patients) | Faeces | 10 tested, 10 positives (100%) | Patient 1: day 3  Patient 2: days 2-19  Patient 3: days 3-11  Patient 4: days 14-17  Patient 5: days 5-15  Patient 6: days 8-15  Patient 7: days 3-10  Patient 8: days 3-10  Patient 9: days 2-14  Patient 10: day 11 | 4 out of 10 (40%) | Patient 4: 9 days  Patient 5: 2 days  Patient 9: 2 days  Patient 10: 10 days |
| **Nicastri et al. [19]** | Italy | 1 | Adult, late 20s | Fever Conjunctivitis | Faeces | 1 tested, 1 positive (100%) | Day 3 of admission | 0 out of 1 | NA |
| **Pan et al. [20]** | China | 17 | Laboratory samples | Unknown | Faeces | 17 tested, 9 positives (53%) | Day 0 to 11 | Not available | Not available |
| **Peng et al. [21]** | China | 9 | Adults | Fever  Respiratory symptoms Digestive symptoms (1 patient) | Anal swab | 9 tested, 2 positives (22·2%) | Patient 1: day 3  Patient 2: unknown | Not available | Not available |
| **Song et al. [22]** | China | 1 | Middle aged female | Respiratory symptoms | Anal swab | 1 tested, 0 positive | NA | NA | NA |
| **Tan et al. [23]** | China | 1 | Male, 73 years | Respiratory symptoms | Rectal swab | 1 tested, 1 positive (100%) | Up to day 23 | 1 out of 1 (100%) | 7 days |
| **Tang et al. [24]** | China | 1 | Male, 10 years | Asymptomatic | Faeces | 1 tested, 1 positive (100%) | 17-25 days after exposure | 1 out of 1 (100%) | 10 days |
| **Wang et al. [25]** | China | 205 | Adults and children, mean age 44 years (range 5 to 67 years) | Unknown | Faeces | 153 tested, 44 positives (29%) | Not available | Not available | Not available |
| **Wu et al. [26]** | China | 74 | Laboratory samples | Fever  Respiratory symptoms  Digestive symptoms | Faeces | 74 tested, 41 positives (55%) | Variable | 32 out of 41 (78%) | Faecal RT-PCR remained positive for a mean duration of 27.9 days which was on average 9.2 days longer than positive NP RT-PCR  Patient 1: 33 days after -ve nasopharyngeal swab  Patient 2: 47 days from symptom onset |
| **Xiao et al. [27]** | China | 73 | Children and adults, 10 months to 78 years old | Unknown | Faeces | 73 tested, 39 positives (53·4%) | 1 to 12 days | 17 out of 39 (23·3%) | Not available |
| **Xing et al. [28]** | China | 3 | Children, 1·5 to 6 years | Fever | Faeces | 3 tested, 3 positives (100%) | Patient 1 and 2: day 4  Patient 3: day 9 (after discharge) | 3 out of 3 (100%) | 8 and 20 days |
| **Xu et al. [29]** | China | 10 | Children, 2 months to 15 years | Asymptomatic  Fever  Respiratory symptoms  Digestive symptoms | Rectal swab | 10 tested, 8 positives (80%) | Patient 1: day 2  Patient 2: day 2  Patient 3: day 3  Patient 4: day 1 of admission  Patient 5: day 1 of admission  Patient 6: day 1 of admission  Patient 8: day 1 of admission  Patient 10: 1 day before symptom onset | 8 out of 8 (100%) | Patient 1: 19 days  Patient 2: 21 days  Patient 3: 21 days  Patient 4: 3 days  Patient 5: 21 days  Patient 6: 19 days  Patient 8: 6 days  Patient 10: 8 days after discharge |
| **Zhang et al. [30]** | China | 23 | Adults, median age 48 years | Unknown | Faeces | 12 tested, 10 positives (83·3%) | Day 4 | 6 out of 10 (60%) | Median duration of positive NP RT-PCR:  10 days  Median duration of positive faecal RT-PCR:  22 days |
| **Zhang et al. [31]** | China | 14 | Adults, median age 41 years (range 18 to 87 years) | Fever  Respiratory symptoms | Faeces | 14 tested, 5 positives (35·7%) | Day 4 to 10 | Not available | Not available |
| **Zhang et al. [32]** | China | 15 | Laboratory samples | Unknown | Anal swab | 15 tested, 4 positives (26·7%) | Day 0 to 5 | Not available | Not available |

NNP: Nasopharyngeal; NA: Not applicable; RT-PCR: Reverse Transcriptase Polymerase Chain reaction

P: Nasopharyngeal; NA: Not applicable; RT-PCR: Reverse Transcriptase Polymerase Chain reaction

**Overview of data extracted from studies included in review [7-32]**
