# Supplementary material for: Persistent viral shedding of SARS‐CoV‐2 in faeces – a rapid review
Source: Colorectal Dis. 2020 Jun 4;22(6):611–20. doi: 10.1111/codi.15138 (PMC7276890; doi:10.1111/codi.15138)
Supplement: Supplementary file 2 — Appendix S1. Supplementary methods. [file CODI-22-611-s002.docx]

**Supplementary methods section**

The Mesh terms used for Medline have already been mentioned in the methods section. The search start date was not specified because the terms ‘COVID-19’, ‘SARS-CoV-2’ and ‘2019-nCoV’ used for Medline being novel, themselves limit the start date. The Lancet Gastroenterology and Hepatology was searched for using the terms ‘COVID-19’, ‘SARS-CoV-2’ and ‘2019-nCoV’ while in all other databases (WHO, GUT, NICE etc.), and medRxiv and bioRxiv preprints, the COVID-19 articles are mentioned in a separate section. These were manually screened by their titles to find the relevant articles for faecal viral shedding or gastrointestinal symptoms in COVID-19 patients.

Search for Twitter by SS done on 08.04.2020 was as follows:

1. Terms were entered into Twitter search bar

- Terms entered in each separate search were: “shedding faeces cov”, “shedding cov”, “shedding faeces corona” “shedding corona”, “shedding stool cov”, “shedding stool corona”

2. “top” tweets options selected, and all tweets returned in search were read to look for relevant content

- Tweets were read and those that included a link to an article relating to the search term were selected and links to article clicked
- “latest” tweets option selected, and all tweets returned in search were read to look for relevant content
- Tweets were read and those that included a link to an article relating to the search term were selected and links to article clicked

Identified articles were then sent to the core term (SG, JP) and screened for relevance.

Links for accessing the various databases are provided in the following table:

| **Database** | **Access links** |
| --- | --- |
| WHO database | <https://search.bvsalud.org/global-literature-on-novel-coronavirus-2019-ncov/> |
| NICE | <https://www.nice.org.uk/guidance/published?type=cov,coa> |
| CEBM | <https://www.cebm.net/oxford-covid-19-evidence-service/> |
| Lancet Gastroenterology and Hepatology | <https://www.thelancet.com/action/doSearch?searchType=quick&searchText=COVID-19&searchScope=series&journalCode=langas&seriesISSNFltraddfilter=2468-1253&occurrences=all&code=lancet-site&journalCode=langas> |
| NEJM | <https://www.nejm.org/coronavirus?query=main_nav_lg> |
| GUT | <https://gut.bmj.com/pages/covid-19/> |
| Gastroenterology | <https://www.gastrojournal.org/content/covid19> |
| medRxiv and bioRxiv preprints | <https://www.medrxiv.org/> |
| AJG | <https://journals.lww.com/ajg/Pages/collectiondetails.aspx?TopicalCollectionId=3> |
